# Early tibial loosening in the Attune total knee arthroplasty system: a retrieval analysis of clinical, demographic, and implant-related factors

**DOI:** 10.1007/s00590-025-04514-y

**Published:** 2025-10-17

**Authors:** Amir Reza Ghasemi, Bastian Kreilmann, Alan Kop, Alfredo Pineda, Marcel Dudda, Monika Herten, Moreica Pabbruwe

**Affiliations:** 1 Department of Trauma, Hand and Reconstructive Surgery, University Hospital Essen, University of Duisburg-Essen, Essen, Germany; 2 Dr. Ghasemi & Colleagues, Düsseldorf, Germany; 3 Royal Perth Hospital, Perth, Australia

**Keywords:** Total knee arthroplasty, Retrieval analysis, Aseptic loosening, Tibial baseplate, Attune, Total knee arthroplasty, Attune, Tibial loosening, Cement adhesion, Implant failure, Polyethylene oxidation

## Abstract

**Background:**

Early tibial loosening has been reported in the Attune total knee arthroplasty (TKA) system, with uncertainty regarding the contribution of clinical, demographic, and implant-related factors.

**Methods:**

We retrospectively analyzed 130 retrieved Attune TKAs from 126 patients across nine institutions. Clinical and demographic data, reasons for revision, and implant features were collected. Cement coverage of 96 cemented tibial baseplates was quantified using digitized image analysis, and 15 polyethylene tibial bearings underwent mechanical and oxidation testing. Statistical significance was defined as *p* < 0.05.

**Results:**

The mean time in situ was 2.0 years (range 0.1–8.3). Main reasons for revision were loosening (37.7%), infection (31.5%), and pain (23.1%). Male sex was associated with higher risk of infection (OR 3.44; 95% CI 1.53–7.75; *p* = 0.003), while female sex was associated with increased risk of aseptic loosening (OR 0.375; 95% CI 0.142–0.992; *p* = 0.048). Low cement coverage was significantly associated with aseptic loosening (*p* = 0.003). S+ baseplates demonstrated superior cement adhesion compared to the primary Attune baseplate (*p* = 0.001). Polyethylene bearings showed no evidence of oxidative degradation (average ketone index 0.47; no white banding observed).

**Conclusions:**

Aseptic tibial loosening in the Attune TKA system is significantly associated with poor cement adhesion, while the redesigned Attune S+ baseplate demonstrated improved cement coverage. No adverse polyethylene degradation was observed. These findings highlight the role of implant design and cement adhesion in early failure and underscore the value of retrieval analysis for improving TKA outcomes.

## Introduction

Total knee arthroplasty (TKA) is one of the most successful procedures in orthopedics, providing reliable pain relief and functional improvement for patients with advanced osteoarthritis. Nevertheless, early failure of TKA remains a significant concern, with aseptic loosening of the tibial component being among the most frequent causes of revision in the early years after implantation [1, 2].

Multiple factors have been implicated in early tibial component failure, including patient-related variables (such as body mass index, bone quality, and activity level), surgical technique (component alignment, cementation technique, and interface contamination), and implant design [3–5].

The Attune knee system (DePuy Synthes, Warsaw, Indiana, USA) was launched in 2011 with the intention of improving pain outcomes, stability, and range of motion compared to earlier designs such as the PFC Sigma. However, soon after its introduction, several case series and retrieval studies reported an unexpectedly high incidence of early tibial loosening at the implant–cement interface [4–6]. These failures were characterized by debonding between the tibial tray and the cement, rather than at the bone–cement interface.

In response to these findings, DePuy introduced the redesigned Attune S+ tibial baseplate in 2017, featuring undercut cement pockets and a micro-blast surface finish intended to enhance cement fixation. Early biomechanical data and recent randomized RSA trials have suggested that the S+ design provides improved cement adhesion and fixation strength compared to the original Attune baseplate [3, 7].

Despite these modifications, there remains limited evidence directly correlating clinical and demographic factors with aseptic tibial loosening in the Attune system. Furthermore, while antioxidant-stabilized polyethylene inserts are a key design feature of the system, few studies have assessed their in vivo mechanical or oxidative performance after implantation.

### Aim of the study

The aim of this study was to investigate early failure mechanisms in retrieved Attune TKAs by analyzing clinical and demographic risk factors for aseptic tibial loosening, comparing cement adhesion between the original Attune baseplate and the redesigned Attune S+, and evaluating the in vivo oxidative and mechanical properties of the antioxidant-stabilized polyethylene bearings.

## Materials and methods

This retrospective retrieval analysis included 130 Attune total knee arthroplasties (TKAs) collected between July 2014 and November 2022 from 126 patients revised by 25 surgeons at nine institutions across Western Australia. Retrievals were accompanied by standardized forms documenting patient demographics, clinical history, implant configuration, and surgeon observations at revision.

### Ethical approval

This study was conducted at the Centre for Implant Technology and Retrieval Analysis (CITRA), Royal Perth Hospital, under the state-wide *Release of Human Tissue and Explanted Medical Devices Policy* (MP0129/20, Department of Health, Western Australia). This policy provides the mandatory ethical and legal framework for the collection and analysis of explanted medical devices across Western Australia. Accordingly, no additional institutional review board approval was required.

### Retrieval analysis

All retrieved components were visually examined and further assessed under a stereomicroscope (Leica MZ10, Germany) at up to 60× magnification. Patient data included age, sex, BMI, activity level, reason for revision, and implant type. Photographs of the tibial baseplate undersurface were obtained for cement coverage analysis, and polyethylene inserts were assessed for wear and surface degradation.

### Cement coverage assessment

Cement adhesion on cemented tibial baseplates (n = 96) was quantified using digitized photographs and analyzed with ImageJ software (National Institutes of Health, Bethesda, MD). For each baseplate, the percentage of cement adhesion across the undersurface was calculated, with 0% indicating no visible cement and 100% indicating complete coverage. Two independent observers performed the analysis, and discrepancies greater than 5% were resolved by consensus. Inter-observer reliability for cement coverage was excellent (intraclass correlation coefficient > 0.90).

### Activity level

Patient activity level was determined by the treating surgeon prior to revision surgery. It was categorized as low for patients who were elderly or low-demand, requiring walking aids and able to walk for only 0–15 min; moderate for patients not using mobility aids, capable of performing normal activities of daily living and intermittent exercise; and high for patients without mobility aids who regularly exercised or worked in physically demanding occupations, and were able to walk for more than 1 h without limitation.

### Polyethylene analysis

A subset of 15 tibial inserts underwent mechanical and oxidative testing. Specimens were cleaned, rinsed in distilled water, and stored at –5 °C until analysis. Oxidative degradation was evaluated using Fourier transform infrared (FTIR) spectroscopy in accordance with ASTM F2102 [8], with a ketone index (1715 cm⁻^1^/1370 cm⁻^1^) > 1.2 considered indicative of oxidation. Mechanical properties were tested using small punch testing according to ASTM F2183 [9] on an Instron 5566 system.

### Statistical analysis

All analyses were performed using R statistical software (R Foundation for Statistical Computing, Vienna, Austria). Statistical significance was defined as *p* < 0.05. Continuous variables were compared using the Wilcoxon rank-sum test and categorical variables using the chi-square test. Odds ratios (OR) with 95% confidence intervals (CI) were calculated for risk factor analyses.

## Results

A total of 126 patients (130 TKAs) were included in the analysis. Patient demographics are summarized in Table 1. The cohort consisted of 68 females and 58 males, with a mean age of 69 years (range 49–88) at the time of revision. The mean BMI was 29.4 kg/m^2^ (range 21.2–38.6). The median time to revision was 2.9 years (IQR 1.8–4.1).

**Table 1:** Patient demographics (n = 126 patients, 130 TKAs)

Variable	Value
Number of patients	126
Number of TKAs	130
Female: Male	68:58
Mean age at revision (years)	69 (range 49–88)
Mean BMI (kg/m^2^)	29.4 (range 21.2–38.6)
Median time to revision (years)	2.9 (IQR 1.8–4.1)

### Reasons for revision

The most common reason for revision was aseptic tibial loosening, accounting for 51% of cases (66/130). Infection was identified in 22% (28/130), instability in 15% (20/130), stiffness in 7% (9/130), and other causes in 5% (7/130). The distribution of revision indications is shown in Table 2.

**Table 2:** Reasons for revision (n = 130 TKAs)

Reason for revision	n	%
Aseptic tibial loosening	66	51
Infection	28	22
Instability	20	15
Stiffness	9	7
Other	7	5

### Sex-specific associations

Male sex was associated with higher odds of revision for infection (OR 3.44, 95% CI 1.53–7.75; *p* = 0.003), whereas female sex was associated with lower odds of aseptic tibial loosening (OR 0.375, 95% CI 0.142–0.992; p = 0.048), indicating higher odds in females. These estimates are provided in Table 3.

**Table 3:** Sex-specific odds of revision indications (univariable logistic regression)

Outcome	Exposure (sex)	OR (95% CI)	*p*-Value	Model notes
Infection	Male vs. female	3.44 (1.53–7.75)	0.003	Univariable logistic regression
Aseptic tibial loosening	Male vs. female	0.375 (0.142–0.992)	0.048	Univariable logistic regression

### Cement coverage and aseptic loosening

**Fig. 1:**
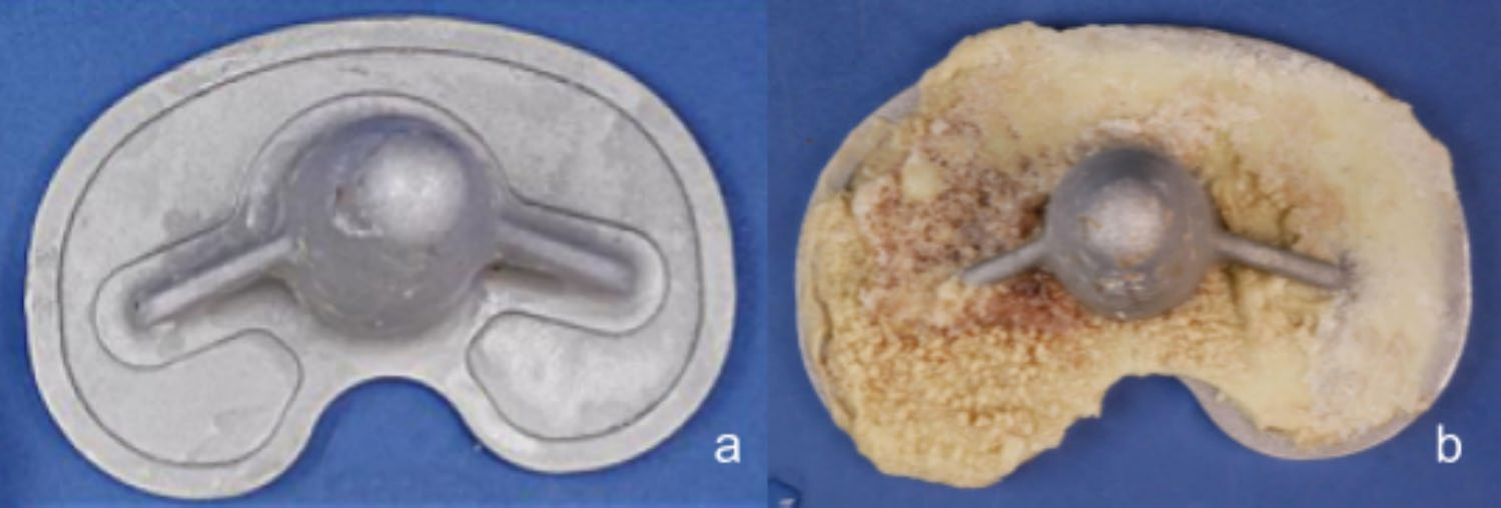
Undersurface of tibial baseplate Attune standard (**a**) and S+ (**b**)

Among the 96 cemented baseplates available for analysis, 48 implants demonstrated low cement coverage (< 50%) and 48 showed high coverage (≥ 50%). Aseptic loosening occurred in 79% of implants with low cement coverage compared with 38% of those with high coverage. Statistical analysis confirmed a significant association, with low cement coverage being strongly correlated with aseptic loosening (OR 3.6, 95% CI 1.8–7.1, *p* = 0.001) (Table 4, Fig. 1).

**Table 4:** Cement coverage and risk of aseptic loosening (n = 96 cemented baseplates)

Cement coverage	n	Aseptic loosening (%)	OR (95% CI)	*p*-Value
< 50% (low coverage)	48	38 (79%)	3.6 (1.8–7.1)	0.001
≥ 50% (high coverage)	48	18 (38%)	Reference	–

**Fig. 2:**
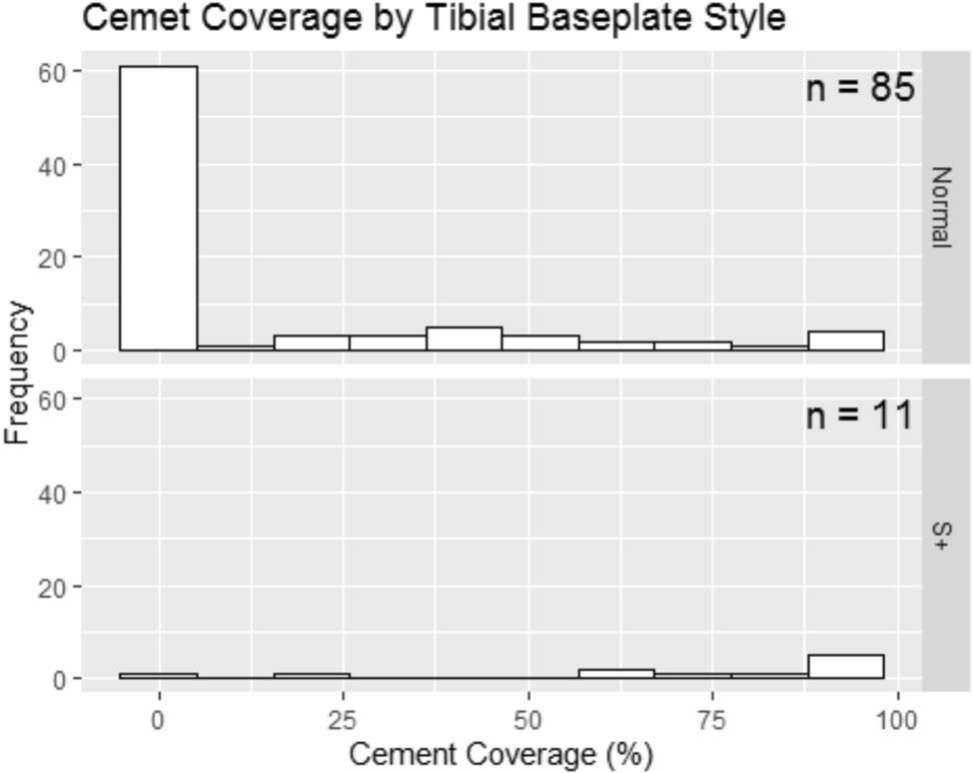
Cement coverage (%) of the tibial baseplate undersurface by design: standard Attune versus Attune S+ (Wilcoxon rank-sum test, *p* = 0.001)

When comparing implant designs, the S+ baseplates demonstrated markedly higher cement coverage than the standard Attune tibial baseplates. The majority of standard designs showed minimal adhesion, whereas the S+ variants exhibited nearly complete cement coverage. This difference was statistically significant (*p* = 0.001, Wilcoxon rank-sum test) (Fig. 2).

### Activity level and implant survival

Activity level prior to revision was determined by the treating surgeon and classified as low, moderate, or high. Patients in the high-activity group were predominantly younger, physically active individuals (e.g., manual workers, athletes). No significant correlation was found between pre-revision activity level and risk of aseptic loosening (*p* > 0.05).

### Polyethylene (PE) wear and backside analysis

PE inserts from revised TKAs were examined for evidence of wear or backside damage. No cases of severe wear were identified, and backside damage was minimal across all implants. These findings suggest that PE-related failure mechanisms were not a major contributing factor to early revision in this cohort. Mechanical and oxidation test results are summarised in Table 5.

### Other implant characteristics

No significant differences were observed between fixed vs. mobile bearings or between CR and PS designs with respect to time in situ or risk of aseptic loosening (data not shown).

## Discussion

The present retrieval analysis demonstrates that aseptic tibial loosening was the most frequent cause of early revision in the Attune TKA system, accounting for more than half of all cases. This finding is consistent with recently published data reporting a high incidence of radiolucent lines and loosening in cemented Attune tibial components [1, 2]. Our study further confirms that loosening was strongly associated with inadequate cement coverage of the tibial baseplate undersurface, whereas implants with higher cement adhesion were less frequently revised for loosening.

In particular, we observed a marked difference between the standard Attune tibial baseplate and the later S+ design. The S+ variant demonstrated substantially greater cement adhesion, which was statistically significant and may explain the reduced loosening risk observed in this group. This is in line with recent randomized and radiostereometric (RSA) studies that reported improved fixation and migration profiles in enhanced fixation designs compared with traditional tibial components [3, 10]. Taken together, these data suggest that baseplate design modifications that optimize cement interdigitation may play a critical role in preventing early loosening. Importantly, there is ongoing debate as to whether current TKA implants, including newer design variants, are adequately tested and validated for personalized alignment strategies [11]. This broader regulatory perspective highlights the need for further scrutiny when extrapolating design improvements to diverse surgical philosophies.

Other potential mechanisms for early revision, such as polyethylene wear or backside damage, were not observed in our cohort. Similarly, pre-revision activity level did not correlate with an increased risk of tibial loosening. While previous studies have emphasized the role of patient-related factors, such as activity and alignment, in implant longevity [12], our findings suggest that inadequate cement fixation rather than patient activity was the primary driver of early failure in this series. Notably, dynamic RSA studies have shown that mobile-bearing PS designs may permit wider translations and rotations compared with fixed-bearing implants [13]. Although our cohort did not demonstrate significant differences between bearing types, these biomechanical findings should be considered when interpreting implant survivorship in larger populations.

This study has several limitations. First, its retrospective design inherently carries risk of bias. Second, the number of S+ baseplates was relatively small compared to the standard design, which may limit the statistical power of subgroup analyses. Third, activity level was determined clinically rather than with a validated scoring system, and measurement accuracy of cement coverage, although standardized, may still involve observer variability. Finally, as a single-center retrieval study, generalizability to other populations may be limited. Nevertheless, this analysis represents one of the largest retrieval cohorts of Attune TKAs to date, providing valuable insights into implant fixation.

From a clinical perspective, our findings underline the importance of meticulous cementation technique and consideration of implant design in primary TKA. The observation that the S+ baseplate offers superior cement fixation has direct surgical relevance and may help reduce the risk of early tibial component loosening.

**Table 5:** Mechanical and oxidation test results for explanted polyethylene inserts

Lab No	Sub Style	Implant Classification	Time In Situ (years)	Work To Failure (mJ)	Elastic Modulus (MPa)	Load At Break (N)	KI	White Banding
8557		FIXED BEARING; PS (Posterior Stabilized)	0.1	253	757	89		Not Measured
8365		CR (Cruciate Retaining); FIXED BEARING	0.1	236	746	84.1		Not Measured
9044		CR (Cruciate Retaining); FIXED BEARING	0.4	232				Not Measured
9456		CR (Cruciate Retaining); FIXED BEARING	0.6	228	579	83		Not Measured
15,572	RP (Rotating Platform)	MOBILE BEARING; PS (Posterior Stabilized)	1	282	1099	93.6	0.5	No
10,545	RP (Rotating Platform)	MOBILE BEARING; PS (Posterior Stabilized)	2	212.3	885.6	81.6		Not Measured
10,633		CR (Cruciate Retaining); FIXED BEARING	2	246.7	705.4	87.6	0.25	No
11,248		FIXED BEARING; PS (Posterior Stabilized)	4.4	242.2	854.5	89.7	0	No
11,741		CR (Cruciate Retaining); FIXED BEARING	4.7	243	968.8	87.4	0.55	No
13,508	RP (Rotating Platform)	MOBILE BEARING; PS (Posterior Stabilized)	5.6	288.1	682.8	89.7	0.6	No
14,469		FIXED BEARING; PS (Posterior Stabilized)	5.8	244.5	940.8	88	0.8	No
12,539	RP (Rotating Platform)	CR (Cruciate Retaining); MOBILE BEARING	6	253.8	872.2	89.9		No
13,481		CR (Cruciate Retaining); FIXED BEARING	6.7	283.7	737	93.7	0.5	No
15,166		MOBILE BEARING; PS (Posterior Stabilized)	8.2	237	1059	87.8	0.4	No
14,075		CR (Cruciate Retaining); FIXED BEARING	8.3	0	0	0	0.75	No
14,401	RP (Rotating Platform)	CR (Cruciate Retaining); MOBILE BEARING	8.3	247.2	941.8	88.1	0.3	No

## Conclusion

The present retrieval analysis confirms that aseptic tibial loosening remains the predominant cause of early failure in the Attune TKA system and is strongly associated with inadequate cement adhesion at the tibial baseplate–cement interface. The redesigned S+ baseplate demonstrated significantly improved cement coverage, which may translate into reduced loosening risk. Polyethylene analysis revealed no evidence of oxidative degradation, indicating that PE-related mechanisms did not contribute to early revision in this cohort. These findings emphasize the critical role of cementation quality and implant design in preventing early failure. Ongoing evaluation of fixation strategies, particularly in the context of evolving alignment philosophies, will be essential to optimize TKA survivorship.

## Data Availability

No datasets were generated or analysed during the current study.
